# An application of kernel methods to variety identification based on SSR markers genetic fingerprinting

**DOI:** 10.1186/1471-2105-12-177

**Published:** 2011-05-20

**Authors:** Florian Martin

**Affiliations:** 1 Biological Systems Research, Philip Morris International R&D, Philip Morris Products S.A., Neuchâtel, Switzerland

## Abstract

**Background:**

In crop production systems, genetic markers are increasingly used to distinguish individuals within a larger population based on their genetic make-up. Supervised approaches cannot be applied directly to genotyping data due to the specific nature of those data which are neither continuous, nor nominal, nor ordinal but only partially ordered. Therefore, a strategy is needed to encode the polymorphism between samples such that known supervised approaches can be applied. Moreover, finding a minimal set of molecular markers that have optimal ability to discriminate, for example, between given groups of varieties, is important as the genotyping process can be costly in terms of laboratory consumables, labor, and time. This feature selection problem also needs special care due to the specific nature of the data used.

**Results:**

An approach encoding SSR polymorphisms in a positive definite kernel is presented, which then allows the usage of any kernel supervised method. The polymorphism between the samples is encoded through the Nei-Li genetic distance, which is shown to define a positive definite kernel between the genotyped samples. Additionally, a greedy feature selection algorithm for selecting SSR marker kits is presented to build economical and efficient prediction models for discrimination. The algorithm is a filter method and outperforms other filter methods adapted to this setting. When combined with kernel linear discriminant analysis or kernel principal component analysis followed by linear discriminant analysis, the approach leads to very satisfactory prediction models.

**Conclusions:**

The main advantage of the approach is to benefit from a flexible way to encode polymorphisms in a kernel and when combined with a feature selection algorithm resulting in a few specific markers, it leads to accurate and economical identification models based on SSR genotyping.

## Background

Genetic markers are target sites in the genome that differ between individuals of a population. These differences can occur in DNA that codes for specific genes, or usually in the vast areas of intergenic DNA. These differences in the make-up of the genetic content at a specific site in the genome are often referred to as polymorphisms (literally "multiple forms"). These polymorphisms are detected with a range of different technologies of which simple sequence repeat markers (SSRs) [1] and single nucleotide polymorphisms (SNPs) are currently the most commonly used types. The markers used in this study are SSRs. The SSRs of interest for marker development include di-nucleotide and higher order repeats (e.g. (*AG*)*_n_*, (*TAT *)*_n_*, etc.). The number of repeats usually ranges between just a few units to several dozens of units. The polymorphism can exist at a locus containing a microsatellite between individuals of a population and is characterized as a different number of repeat units of the microsatellite, which is reported by several authors to result from an unbiased single-step random walk process [2,3].

The detection of these differences occurs by site-specific amplification using polymerase chain reaction (PCR) [4] of the DNA followed by electrophoresis in which the DNA fragments are essentially separated by size. Fragment sizes at a specific locus in the genome are also referred to as "alleles". Depending on the ploidy level of the organism being studied (haploid, diploid, tetraploid), an individual can have one or more alleles at a specific locus. The set of alleles that has been collected for a given individual (often representing a single sample in the study) is referred to as the "genotype" of that individual.

The polymorphism within a population can serve different purposes [5-7]: marker assisted selection in plant breeding [8], genome selection during gene introgression in plant breeding [9], genome mapping [10-12], gene tagging [13], population genetic structure [14,15], and cultivar identification [16-20].

Our purpose is to propose an approach for using SSR marker genotypes to build predictive models to identify commercial tobacco varieties. Predicting unknown samples requires genotyping. When large numbers of samples and SSR markers are involved, the genotyping process can be costly in terms of laboratory consumables, labor and time. As a consequence, it generally makes sense to select a minimal set of markers to build the prediction model.

As mentioned above, primers associated with an SSR marker that are amplified by PCR on a DNA sample lead to several amplicon sizes, (the "alleles") defining the genotype of the sample. The results of such amplification on one sample are of the form *g*_1 _= *a*_1_/*a*_2_/.../*a_m _*where *a_i _*is an integer depending on the number of microsatellite repeats between the two flanking primers and *m *depends on the ploidy type of the organism from which the DNA is extracted (it can vary from one to several). For SSR markers, the number *a_i _*is qualitative only and not quantitative as (*a_i_*, *a_i _*+10) is no more different than (*a_i_*, *a_i _*+ 2) from the point of view of the genetics. A snapshot of such a dataset is given in Table 1.

**Table 1:** Sample of SSR genotyping data

	SSR1	SSR2	SSR3	SSR4	...
Sample 1	177/181	191/193	172	176/182/186	...
Sample 2	177/181	-	172/174	176	...
Sample 3	175/177	193	168/172	180/182	...

The challenge in building a supervised prediction model is therefore to handle these data, which are neither continuous, nor nominal, nor ordinal. A straightforward approach would be to code all the alleles and treat the 0 - 1 data in the feature space whose dimensions are defined by the distinct alleles in the training set. However, unless the initial feature space is enriched with extra dimensions and the prediction model is retrained, metrics on this binary data space will not take into account new alleles coming from new samples in order to use a prediction model built on this feature space of fixed dimension. Defining the feature space as the infinite (countable) direct sum of {0, 1} spaces and the usage of a kernel overcomes this limitation.

Geneticists ususally compute the Nei-Li distance [21] to estimate the evolutionary distance between the samples and unsupervised methods, like hierarchical clustering or principal coordinate analysis on the Nei-Li distance matrix, are commonly used to treat SSR data; but those are not suited to predict new DNA samples. To our knowledge, only Artificial Neural Networks have been used in a supervised manner in this context [22], where the allele binary coding was used.

The purpose of this article is twofold:

1) show that encoding the SSR marker polymorphism into the Nei-Li similarities indeed defines a positive definite kernel that will allow the usage of supervised methods to address specific discrimination tasks;

2) describe a simple filter method [23] for selecting identification kits, consisting of a small number of SSR markers that have acceptable discrimination ability for a specific task.

## Results and Discussion

In this study, *Nicotiana tabacum*, a functional diploid was used. The methods described above will be applied to four datasets, with distinct discrimination purposes. The material and method description for the primers development and genotyping of the samples can be found in [24]. Four datasets were developed:

a) tobType: A set of 91 varieties were genotyped on 186 SSR markers without replicates; that lead to 91 observations (see additional file 1). The objective is to discriminate the following tobacco types: Burley, Flue Cured and Oriental.

b) landRace: A set of 10 different landraces of a given variety (5 plants with 5 replicates) were genotyped on 19 SSR markers for a total of 250 observations (see additional file 2). The groups to discriminate are the 10 landraces of this variety.

c) geoVar: A set of 67 different varieties from the same geographic region were genotyped on 48 SSR markers for a total of 93 observations (see additional file 3). The objective is to discriminate the 12 known subtypes.

d) ORvar: A set of 38 different varieties from the same tobacco type (oriental) were genotyped on 48 SSR markers for a total of 88 observations (see additional file 4). The objective is to discriminate 8 pre-defined families.

Mutual Information based Feature Selection (MIFS) [25] and maximum Relevance - Minimum Redundancy (mRMR) [26] and our method (the naive case *α *= 0 and the cases *α *> 0) are compared on those four datasets, generated internally. The comparison is done on a range from *N *= 2 to 8 markers. For MIFS, the additional parameter *β*[25] (which balances the importance and the complementarity of a feature) is chosen by cross-validation over the set of values 0, 0.75, 1, 1.25 and for our method *α *is chosen over the same set. The cross-validation loop includes the feature selection to avoid a possible selection bias. The results shown in the tables are the best 10-fold cross-validated results over the parameters of each method and the classification error rates for the different kit sizes, when combined with kernel linear discriminant analysis (KLDA) or kernel principal component analysis followed by linear discriminant analysis (KPCLDA) are shown in Table 2 and Table 3. The number of markers in the kit, *N*, is kept as a separate parameter as a consensus between performance and kit size has to be reached.

**Table 2:** KPCLDA cross-validation results

KPCLDA	N = 2	N = 3	N = 4	N = 5	N = 6	N = 7	N = 8
tobType							

FS	0.02	**0.01**	**0.00**	**0.00**	**0.00**	**0.00**	**0.00**
FS, *α *= 0	0.02	0.02	**0.00**	**0.00**	0.01	0.01	**0.00**
MIFS	**0.01**	**0.01**	0.01	**0.00**	**0.00**	**0.00**	**0.00**
mRMR	0.16	0.06	**0.00**	**0.00**	**0.00**	**0.00**	**0.00**

landRace							

FS	0.40	0.19	0.11	0.08	**0.03**	**0.02**	**0.03**
FS, *α *= 0	0.43	**0.18**	**0.10**	0.08	0.05	0.03	0.04
MIFS	**0.36**	0.19	0.16	0.14	0.09	0.04	**0.03**
mRMR	**0.36**	0.19	0.11	**0.07**	0.06	0.06	0.04

geoVar							

FS	0.35	0.25	0.24	0.22	**0.14**	0.21	**0.15**
FS, *α *= 0	0.37	0.29	0.31	0.28	0.19	**0.18**	**0.15**
MIFS	**0.33**	0.31	0.26	0.19	0.19	0.26	**0.15**
mRMR	0.35	**0.24**	**0.22**	**0.13**	0.20	**0.18**	0.21

ORvar							

FS	**0.14**	0.13	**0.08**	**0.09**	**0.06**	**0.07**	**0.03**
FS, *α *= 0	0.29	0.16	0.17	0.11	0.14	0.12	0.08
MIFS	0.19	**0.12**	0.11	0.14	0.11	0.18	0.09
mRMR	0.26	0.13	0.09	0.13	0.09	0.06	0.07

**Table 3:** KLDA cross-validation results

KLDA	N = 2	N = 3	N = 4	N = 5	N = 6	N = 7	N = 8
tobType							

FS	**0.01**	**0.01**	**0.00**	**0.00**	**0.00**	0.01	**0.00**
FS, *α *= 0	0.02	**0.01**	**0.00**	0.02	0.01	**0.00**	0.01
MIFS	0.09	0.02	0.03	0.01	0.01	0.02	0.02
mRMR	0.23	0.19	0.06	0.08	0.04	0.02	0.04

landRace							

FS	0.36	0.16	**0.06**	**0.03**	**0.02**	**0.00**	**0.00**
FS, *α *= 0	0.40	0.16	0.07	0.04	0.03	0.01	0.01
MIFS	**0.35**	**0.13**	0.11	0.09	0.04	0.02	0.01
mRMR	0.36	**0.13**	0.11	0.05	**0.02**	0.02	0.01

geoVar							

FS	0.36	**0.20**	**0.23**	0.18	**0.15**	0.17	**0.16**
FS, *α *= 0	0.35	0.33	0.31	0.20	0.16	**0.14**	0.18
MIFS	0.36	0.26	0.25	0.21	0.20	0.22	0.17
mRMR	**0.31**	0.28	0.25	**0.14**	0.17	0.16	**0.16**

ORvar							

FS	**0.15**	0.12	0.09	**0.09**	**0.05**	**0.05**	**0.03**
FS, *α *= 0	0.27	0.14	0.11	0.13	0.11	0.07	0.10
MIFS	0.19	0.12	0.14	0.12	0.11	0.14	0.06
mRMR	0.24	**0.10**	**0.08**	**0.09**	0.07	0.08	0.07

Overall, the proposed method leads to satisfying results, comparable or better than the other ones. Only in four cases (both classification methods confounded), improved performance by at least 3% lower error rate were found by the other selection methods. Out of 56 cases, the proposed method obtained the best results (equal or better to the compared methods) in 42 cases. Though generally the improvements are slight, for a few cases the relative difference in error rates is substantial.

It is interesting to consider the case *α *= 0 separately as it forbids skipping features and allows an evaluation of the benefit of skipping markers. In the vast majority of comparisons, skipping markers is beneficial and the differences in error rate range from 1% to 15% (*ORvar *dataset, *N *= 2).

Comparing the obtained results on all three methods to the classification error rates using all the markers (see Table 4), one can observe that better error rates can be obtained by the selected kits for all the datasets except the *geoVar *dataset, where only KPCLDA with 6 markers can almost reach the error rate of the full set of SSR.

**Table 4:** Cross-validation results using the full set of markers

Dataset	KPCLDA	KLDA
tobType	0 +/- 0	0.065 +/- 0.029
landRace	0.117 +/- 0.012	0 +/- 0
geoVar	0.132 +/- 0.041	0.081 +/- 0.04
ORvar	0.069 +/- 0.044	0.098 +/- 0.043

In order to evaluate how the selected set of markers performs versus the other subsets of cardinality 5, an exhaustive search (11'628 possibilities) was performed. The 10-fold cross-validation results from this simulations are summarized in Table 5. Among all the subsets of size 5, the one chosen by the algorithm belongs to the best 0.5% sets of size 5 for KPCLDA (best selected kit error rate = 7% (mRMR) - 8% (FS), best subset = 4%) and the best 0.5% for KLDA (best marker error rate = 3% (FS), best subset = 1%), which show the abiltiy of the feature selection algorithm to capture the few most important markers.

**Table 5:** Simulation results

Quantiles	KPCLDA	KLDA
0%	0.04	0.01
0.5%	0.08	0.03
1%	0.10	0.04
5%	0.15	0.08
25%	0.23	0.14
50%	0.31	0.20
75%	0.38	0.25
100%	0.78	0.62

Summary of the cross-validation results for all possible combinations of 5 markers among 19 markers (*landRace *dataset)

The final kit sizes retained for the datasets under consideration are 3 for *tobType*, 5 for *landRace*, 5 for *geoVar*, 6 for *ORvar*. For the 2 first datasets no marker is skipped, for the third dataset the fifth most powerful marker is skipped and finally for the fourth dataset the third and fourth most powerful markers are skipped by the algorithm.

## Conclusions

The Nei-Li similarity was shown to define a positive definite kernel on the set of marker genotypes and therefore is a very convenient way to encode the polymorphisms contained in SSR marker data. It has shown its ability to be used further for SSR fingerprint based predictions. To our knowledge, the usage of kernel methods in this context is new.

On the four case studies presented, the proposed algorithm for selecting SSR marker kits can definitely lead to economical and efficient prediction models for discrimination. The algorithm is independent of the supervised method chosen in the modelling process (so-called filter method).

The results also show that as a general rule, the full set of markers is not necessarily the most predictive kit, and for all case studies presented, similar classification performance can be achieved with less than 8 markers.

Simulation studies show that the kit selection algorithm performs well as compared to the best subset selection when combined with KLDA or KP-CLDA; both methods leading to low classification error rate. Feature selection strategies that can deal with categorical data in classification are not so common and the proposed filter approach might be useful in other contexts as well.

The main advantage of the approach is to benefit from a fast algorithm that results in a few specific markers for a given task. An exhaustive search is generally infeasible or is very time consuming. The choice of the constant can be done by cross-validation. However, from our experience *α *= 1, is consensually a good default choice and performs well.

The choice *α *= 0 (i.e, no consideration for the redundancy) leading to a very straightforward approach, is usually less performant; even though it leads to the best results in 9 cases. Hence, this possibility should not be disregarded when performing a cross-validation experiment on *α*.

When the number of markers becomes smaller, the missgenotyping effect becomes more pronounced and new genotypes on new measured samples affect the genetic dissimilarities more (even with a smaller proportion of prototypes). Therefore, it should be stressed that choosing the minimum number of markers for a given problem can lead to weaker generalization properties of the classifier due to the fact that the new samples whose type or landraces are unknown are perhaps not in the original dataset and may have new genotypes. It is therefore recommended, in practice, to use at least 5 markers in a selection kit, if the number of classes to discriminate is greater than 4. Moreover, the pre-processing and identification of the electrophoresis amplicons as well as the marker usage have to be well established in order to test new samples. The quality of the laboratory work and of the SSR markers development used here also contributed to the efficiency of the models.

## Methods

### Kernel methods for genotyping data

As mentioned in the introduction, genotyping data are neither continuous, nor nominal, nor ordinal. Considering the allele (and not genotype) data as nominal and using a 0 - 1 coding can be done but is not without problems.

The difficulty in using this special type of data is discussed in [27], where the authors argue against the use of Fisher Discriminant Analysis due to the discrete nature of the data, preferring the usage of Artificial Neural Networks based on the allele frequencies. A possible way to handle the binary data is to build a model using the DISQUAL approach as presented in [24,28]. Despite the presence of Multiple Correspondence Analysis (which is intended to make the model more robust), this approach is rather sensitive to genotyping error (misassignment of alleles).

Indeed, the natural binary coding feature space whose dimensions are the alleles in the training set ({0, 1}*^N ^*where *N *is the number of distinct alleles in the training set) is not the best option because, for a given SSR marker, the alleles obtained on new samples can often be lacking in the original training set. Therefore, any metric on {0, 1}*^N ^*cannot use the information in the newly detected alleles in a prediction model built on the original 0 - 1 space of fixed dimension and may lead to erroneous results. To avoid this issue, one should rather consider an infinite dimensional 0 - 1 coding in  where ℕ ≅ ℕ*^m ^*dimensions represent all the possible alleles of *m *markers. This consideration makes the use of kernels and kernel methods well suited in this context.

Geneticists usually estimate the degree of polymorphism between two sample genotypes by computing the Nei-Li genetic distance between them. The similarity associated with that distance will be shown to define a positive definite kernel on the set of the genotyped samples. Hence, this kernel will be our prefered choice.

Given two samples *S*_1_, *S*_2 _on which *m *SSR markers are amplified, leading to *m *genotypes for the first sample  and *m *genotypes for the second sample , where  is seen as the amplicons set, the *Nei-Li genetic distance *between *S*_1 _and *S*_2_, is computed as

Where Δ denotes the symmetric difference of the two sets and | ...| the set cardinality.

This approach overcomes the issues mentioned above as new alleles will be implicitly used in the computation of the Nei-Li distance. Moreover, it is well suited to these data due to their biological meaning and is coherent with the fact that the set of genotypes is partially ordered by set inclusion:  if and only if {*a*_1_,..., *a_n_*} is contained in , which reflects the biological comparison of genotypes. Therefore, given a data set of samples on which *m *SSR markers are amplified, it leads to a dissimilarity matrix whose entries are the estimated genetic distance between a pair of samples. The purpose here is not to accurately estimate the evolutionary distances between the varieties (as those distances are supposed to, see [21]) but to exploit the polymorphism encoded in the SSR data in a meaningful way.

The basic concept of kernel discrimination methods is to model a classifier in a feature space (which will be a Hilbert space) based only on a"similarity" matrix which is assumed to be positive definite. Indeed, if the measure of similarity between the samples is a positive definite kernel [29,30], then classifiers can be trained in the reproducing Hilbert space associated with it [30]. It turns out that the Nei-Li similarity defines a positive definite kernel.

**Lemma **1 - *δ_NeiLi _defines a positive definite kernel over the set of genotypes associated to SSR markers*.

**Proof **Let *S*_1_, *S*_2 _be two samples genotyped and let us consider them as binary vectors in .

Then 1 - *δ_NeiLi _*can be rewritten as . Using the fact that a pointwise product of positive definite kernel is also positive definite (see e.g. [29]), it is sufficient to show that  is positive definite. Now let us define a mapping from  into *L*^2^(ℝ^+^), by ϕ: *S *↦ (*t*↦*e*^-||*S*||*t*^). Now

which proves the lemma.

Once this valid kernel is defined, a wide range of supervised methods can be applied. The supervised approaches investigated in our examples are Kernel-Linear Discriminant Analysis (KLDA, [31]) and Kernel-Principal Component-Linear Discriminant Analysis (KPCLDA, Kernel-Principal Component Analysis followed by Linear Discriminant Analysis as described in [29,32]). To our knowledge kernel approaches have not yet been applied to SSR data.

### Identification kit selection: Discrimination power of a SSR marker

The cost of the SSR analysis is to be taken into account when building a predictive model: the classification results should be obtained with a minimal number of SSR markers in order to be used at a reduced cost.

The exhaustive subset selection is obviously too computer extensive, as subsets of size 5 to 20 should be extracted from hundreds of SSR markers. Hence a strategy has to be developed to address this issue.

As the feature selection in the reproducing kernel Hilbert space associated with our kernel is not useful and by definition of the kernel building, classical embedded method [23] like Lasso [33] or L1-SVM cannot be applied. Therefore, filter methods for SSR selection are natural in our context. Additionally, as the data generated have a long life-cycle and can be used in the long run, the set of markers proposed for a given task is preferred to be independent of the classification method used.

The criteria for having a suitable identification SSR markers kit can be stated as follows: *"Choose the set of markers that show the biggest polymorphism between the groups to discriminate and the lowest polymorphism within the groups"*. This criteria is to be thought of as similar to the famous Fisher's "between/within" maximization criteria used in canonical discriminant analysis.

A score will be computed for each of the SSR markers which represents the ability of a given SSR marker to discriminate between the groups. Additionally, a redundancy score will also be computed in order to assess wether the polymorphism contained in a marker *A *is "similar" to the polymorphism of a second marker *B*. If this is the case, one marker should be dropped in favor of another one explaining a different polymorphism.

Due to the nature of the genotype data, information theoretic measures are well suited here: association between the marker and the group to discriminate is measured through Asymmetric Uncertainty Coefficient [34], which reflects the dependency of the SSR marker and the group to be discriminated and the redundancy between two markers will be quantified by the Uncertainty Coefficient (a normalized version of the mutual information).

For *X *and *Y *two discrete variables, let *H*(*X*) and *H*(*X*, *Y *) denote the entropy and the joint entropy respectively. Empirical estimates are used to evaluate these quantities (, and ).

Following [34], we have:

1) *The symmetric uncertainty coefficient *is defined by . Its asymptotic variance is

2) *The asymmetric uncertainty coefficient *is defined by . Its asymptotic variance is given by

In what follows, the value *U*(*Group|SSR_i_*) ≐ *p_i _*for an SSR marker will be called the discrimination power of the marker for the given group classification, and *U*(*SSR_i_*, *SSR_j_*) ≐ *U_i,j _*will be referenced as the redundancy between the markers *i *and *j*. Therefore a greedy feature selection algorithm can be depicted as follows:

*Sort the discriminating power in decreasing order p*_(1) _≥ ... ≥ *p*_(*m*)_, *and add the marker associated with the p_(1) _to the subset . Now for each following p_(i)_, select marker (i) if the kit size is less than N and if*

*where *0 ≤ *α is a positive parameter controlling the trade-off between power and redundancy*.

The rationale is to keep a discriminating marker (high discriminating power) only if the information encoded in it is not redundant with any of the previous markers in the set. The right-hand side is thought as a heuristic estimate of the multivariate uncertainty Z-score of , which cannot be estimated reliably with a reasonable sample size. The coefficient controls the tradeoff between the discrimination power and the redundancy. This algorithm follow the rationale of both MIFS and mRMR but differs in three ways:

1. It uses the normalized version on the mutual information, the uncertainty coefficients;

2. It leverages the asymptotic variances of those coefficients and therefore enables accounting for the sample variation through the usage of the associated Z-scores;

3. It does not select the feature maximizing a gap between informativeness on the group and the redundancy, but goes in order through the features maximizing the discrimination power and eliminate sequentially the features where the evidence of non-zero redundancy is *α *times lower than the evidence of non-zero discrimination power.

The parameter is set by default to 1, reflecting that a marker is included if the statistical evidence for its discrimination power is bigger than the evidence of its redundancy with the markers already selected. The influence of *α *is one parameter to cross-validate over in the case studies presented with the exception of the simplest case *α *= 0, for which the algorithm is simply choosing the *N *biggest discrimination power.

## Supplementary Material

Additional file 1 **tobType dataset**. A set of 91 varieties were genotyped on 186 SSR markers without replicates; that lead to 91 observations. The objective is to discriminate the following tobacco types: Burley, Flue Cured and Oriental. Click here for file

Additional file 2 **landRace dataset**. A set of 10 different landraces of a given variety were genotyped on 19 SSR markers for a total of 250 observations (5 plants with 5 replicates). The groups to discriminate are the 10 landraces of this variety. Click here for file

Additional file 3 **geoVar dataset**. A set of 67 different varieties from the same geographic region were genotyped on 48 SSR markers for a total of 93 observations. The objective is to discriminate the 12 known subtypes. Click here for file

Additional file 4 **ORvar dataset**. A set of 38 different varieties from the same tobacco type (oriental) were genotyped on 48 SSR markers for a total of 88 observations. The objective is to discriminate 8 pre-defined families. Click here for file
